# Could Mesquite (*Prosopis juliflora*) Help Control Gastrointestinal Parasites in Horses?

**DOI:** 10.3390/ani15091245

**Published:** 2025-04-28

**Authors:** Desiderio Rodriguez Velazquez, Lucrezia Forte, Jorge Antonio Varela Guerrero, Tonantzin Díaz Alvarado, Mona M. M. Y. Elghandour, Aristide Maggiolino, Pasquale De Palo, Abdelfattah Z. M. Salem

**Affiliations:** 1 Facultad de Medicina Veterinaria y Zootecnia, Universidad Autónoma del Estado de México, Toluca C.P. 50000, Estado de México, Mexico; drodriguezv@uaemex.mx (D.R.V.); javarelag@uaemex.mx (J.A.V.G.); tdiaza076@alumno.uaemex.mx (T.D.A.); mmohamede@uaemex.mx (M.M.M.Y.E.); 2 Department of Veterinary Medicine, University of Bari, 70010 Valenzano, Italy; aristide.maggiolino@uniba.it (A.M.); pasquale.depalo@uniba.it (P.D.P.); 3 Dipartimento di Scienze del Suolo, della Pianta e degli Alimenti (Di.S.S.P.A.), Università degli Studi di Bari, Via Giovanni Amendola, 165/a, 70126 Bari, Italy

**Keywords:** equines, *Prosopis juliflora*, alkaloids, anti-parasitics, phytochemicals

## Abstract

Exploring new natural-origin antiparasitic alternatives is essential in addressing the resistance issues that have emerged due to the widespread use of chemical or synthetic antiparasitic compounds. This area remains relatively underexplored despite the increasing recognition of plants like *Prosopis juliflora* (mesquite) for their antiparasitic properties and nutritional value as a high-protein animal feed. This review aims to investigate the antiparasitic potential of *P. juliflora* against gastrointestinal parasites in horses. The review incorporates sources from 2005 to 2024. It was found that various phytochemical compounds from *P. juliflora*, particularly alkaloids, have demonstrated significant antiparasitic activity. Alkaloids are the most studied group due to their antibacterial, antiparasitic, and cytotoxic properties. According to the literature, the phytochemicals produced by *P. juliflora* hold promise as a potential solution for combating gastrointestinal parasites in horses. However, while preliminary findings suggest promising results, in vivo studies are necessary to evaluate the precise antiparasitic efficacy of these compounds and to determine the appropriate IC50 doses of *P. juliflora* alkaloids in horses, as well as to assess potential toxicity and side effects associated with their use.

## Simple Summary

Horses play an important role in transportation, agriculture, and rural livelihoods, especially in developing countries. However, they are often exposed to parasitic infections that harm their health and performance. Overuse of chemical antiparasitic drugs has led to resistance and environmental concerns, making it necessary to explore natural alternatives. This review focuses on *Prosopis juliflora*, a resilient plant rich in bioactive compounds, which has shown promising antiparasitic effects in laboratory studies. Although in vitro results are encouraging, more research is needed to confirm the plant’s effectiveness and safety in live horses.

## 1. Introduction

In many regions, particularly in developing countries and rural areas, a robust horse population contributes to economic and social development by supporting livelihoods through transportation, agriculture, and tourism-related activities [1]. In many developing and underdeveloped regions, equines continue to serve as a vital source of draught power and transportation while also contributing to rural livelihoods through milk production, manure for fertilizer, and, in some cases, cultural and tourism-related roles, thus supporting both the economy and food security of local communities [2,3,4,5,6,7]. However, across all countries, horses—whether used for labor, sport, or leisure—are frequently exposed to numerous risk factors that negatively impact their health [8]. Among these challenges, parasitism stands out as a critical global issue, leading to considerable economic losses within the equine industry [9]. Parasitic infections typically become evident when the host’s immune system is overwhelmed by the parasites. In many cases, equines are owned by resource-constrained farmers who are unable to provide timely treatment for these infections, often resulting in fatalities. The repercussions of such infections include increased management costs, reduced growth, and diminished performance in both sports and labor activities. Parasitized animals face difficulties in nutrient absorption, leading to deficiency-related illnesses, and they may suffer physical complications such as intestinal blockages, allergic reactions, and even death [10].

One of the primary health challenges in equines is gastrointestinal infestation by parasites, including *Strongylus vulgaris*, *Strongylus edentatus*, *Strongylus equinus*, *Parascaris equorum*, *Anoplocephala perfoliata*, and cyathostomins. Various classes of antiparasitic drugs, such as benzimidazoles, tetrahydropyrimidines, and macrocyclic lactones, are commonly employed to combat these infestations [11,12]. However, the frequent and often indiscriminate use of these antiparasitic agents has led to the development of parasite resistance, significantly diminishing their efficacy and effectiveness against these organisms [13]. In addition to resistance, growing concerns have emerged regarding the environmental impact of these drugs, especially macrocyclic lactones such as ivermectin, which are known to persist in the environment and adversely affect non-target invertebrates, including beneficial dung beetles and aquatic organisms. This dual threat—reduced pharmacological efficacy and ecological harm—underscores the urgent need for alternative strategies, such as natural compounds, to regain control over parasitic infestations [14]. Throughout history, various herbs have been widely used in South Asia to address a range of diseases affecting both humans and animals [15]. These medicinal plants hold significant value as they help alleviate the financial strain on farmers and manage allergic reactions effectively [16]. Among these, *Nigella sativa* (*N. sativa*) is one of the most renowned medicinal plants, known for its extensive therapeutic properties, including antibacterial, antiparasitic, antiviral, anticancer, anti-inflammatory, and antidiarrheal effects [17]. Extracts of *N. sativa*—whether aqueous, ethanolic, or oil-based—have demonstrated potent anthelmintic activity against nematode parasites in livestock [18]. Similarly, *Fumaria parviflora* (*F. parviflora*) is a globally recognized medicinal herb traditionally used for treating gastrointestinal disorders. Its anthelmintic potential has been attributed to its phytochemical constituents, such as alkaloids, flavonoids, glycosides, tannins, and fumaricine [19]. Another noteworthy herb is *Flemingia macrophylla* (*F. macrophylla*), which exhibits numerous pharmacological benefits, including antiparasitic effects, due to its bioactive compounds [20]. In addition, *Moringa oleifera*, a plant native to the western regions, is rich in bioactive compounds, particularly in its leaves, where flavonoids, tannins, phenolic acids, saponins, and vitamins contribute to its antioxidant properties. Of these, tannins and saponins are particularly effective against equine gastrointestinal parasites, as they target both larvae and oocytes, making *M. oleifera* a promising natural option for managing parasitic infections in horses [21].

Known for its resilience and ability to thrive in infertile soils due to its nitrogen-fixing capabilities, *P. juliflora* is rich in bioactive compounds with demonstrated antimicrobial, anthelmintic, larvicidal, and insecticidal properties [22]. *Prosopis juliflora* contains a variety of bioactive compounds with antimicrobial, anthelmintic, larvicidal, and insecticidal properties, among others [23]. Additionally, various parts of the tree, such as its leaves and pods, serve as valuable food sources for both humans and animals due to their high energy and protein content, along with significant levels of nutrients, including calcium, phosphorus, and sucrose [24]. Despite its well-documented bioactive properties, the antiparasitic efficacy of *P. juliflora* against gastrointestinal nematodes in horses remains underexplored. Recent studies have shown promising results in vitro, where extracts of *P. juliflora* have demonstrated significant anthelmintic activity against nematodes, including *Strongylus species* and cyathostomins [25]. However, the in vivo efficacy and safety profile of these compounds in equines have yet to be thoroughly investigated. This review aims to focus on the potential of *P. juliflora* as a natural alternative to chemical antiparasitics, particularly against gastrointestinal parasites in horses. We will examine the current literature on its antiparasitic activity, with an emphasis on the phytochemical compounds involved, their mechanism of action, and the need for further studies to confirm their efficacy and safety in equine hosts.

## 2. Methods

To accurately address the potential antiparasitic effects of *Prosopis juliflora* on gastrointestinal parasites in horses, an extensive literature review was conducted using multiple scientific databases, including Scopus, PubMed, and Google Scholar. The bibliographic search covered the period from January 2000 to February 2025. Given the topic, the search strategy was designed to first gather information on the botanical, chemical, and pharmacological properties of *Prosopis juliflora* and then to systematically explore its antiparasitic efficacy, particularly in equine species. The search also encompassed broader contexts, such as the use of plant-based compounds, phytochemicals, and nutraceuticals in veterinary parasitology. The main keywords and keyword combinations used included: “*Prosopis juliflora*” AND (anthelmintic OR antiparasitic OR ovicidal OR phytochemical OR tannin OR bioactive compound), “Mesquite” AND (internal parasites OR gastrointestinal parasites OR equine OR horse OR helminths), “Plant-based anthelmintics” AND (horses OR equids OR livestock), “Phytotherapy” AND “gastrointestinal parasites” AND “horses”, “Tannins” OR “condensed tannins” AND “nematodes” AND “equine”, “Alternative parasite control” AND “horses” AND “medicinal plants”. Boolean operators “AND” and “OR” were applied to maximize the retrieval of relevant articles. No language restrictions were applied during the search process. Inclusion criteria for article selection were relevance to the core topic (i.e., *Prosopis juliflora* and/or plant-based control of gastrointestinal parasites in equids or other livestock); publication date between 2000 and 2025 to include both recent insights and historically significant research; scientific relevance, assessed through article impact, citation count, and presence in peer-reviewed journals. When appropriate, older foundational studies referenced in more recent publications were also included to support background or mechanistic explanations. Experimental trials, in vitro and in vivo studies, reviews, and relevant case studies were all considered. This method ensured a comprehensive and systematic collection of scientific evidence to assess whether *Prosopis juliflora* and its bioactive constituents may represent a viable alternative or complementary strategy for the control of gastrointestinal parasites in horses.

## 3. General Information on Mesquite (*Prosopis juliflora*)

The mesquite (*Prosopis juliflora*) is a deciduous tree or shrub that can reach heights ranging from 2 to 15 m. One of its most notable features, which makes it an excellent option for reforestation, is its ability to thrive in environments where vegetation is typically scarce. It can adapt to areas with low humidity, arid conditions, and soil that is saline, sandy, rocky, or even eroded. This adaptability is largely attributed to its deep root system, which enables it to access nutrients and water from deep within the subsoil [26]. The species is also highly tolerant to varying pH levels, growing optimally in soils with a pH between 6.5 and 8.3, though it has been observed to survive in pH levels as high as 10.4. *P. juliflora* is native to arid and semi-arid regions of Mexico, ranging from Baja California and Chihuahua to Oaxaca and from Tamaulipas to Veracruz. Its range extends to South America, including Peru, North America in Texas, and regions of Africa and Asia [27]. In many of these regions, *P. juliflora* has been classified as an invasive species. In Africa, for example, it has aggressively expanded in countries like Ethiopia, Kenya, and Sudan, leading to conflicts with local farmers due to its displacement of native vegetation and encroachment on agricultural lands. Similarly, in parts of India, the species is referred to as the “Devil Tree” due to its rapid spread and the difficulties in eradicating it [28]. The tree exhibits rapid growth and a high germination success rate, contributing to its swift propagation in areas where it is introduced. However, this rapid spread can be detrimental, as it often displaces native vegetation [26]. The allelopathic properties of *P. juliflora* allow it to inhibit the germination of nearby seeds or damage surrounding plants, likely due to the high solubility of its allelopathic compounds in water [29]. Specific studies have highlighted that the allelopathic effects of *P. juliflora* can significantly reduce the biomass and germination rate of native species such as *Sorghum bicolor* and *Cenchrus ciliaris*. The compound juliprosopine, a phenolic alkaloid isolated from the plant, is suspected to play a central role in this inhibitory effect [30]. These properties exacerbate the ecological challenges in regions where the species is invasive.

A key ecological benefit of *P. juliflora* is its ability to restore fertility to degraded soils, such as those eroded, sandy, or nutrient-poor [31]. By influencing the carbon and nitrogen cycles, this species enhances the soil’s organic matter content and bioavailability of nutrients [32]. As the tree matures, it contributes to increased levels of phosphorus and nitrogen, both in its foliage and the surrounding soil. This nutrient enrichment is further augmented by symbiotic relationships with nitrogen-fixing bacteria [22]. In addition, *P. juliflora* demonstrates significant potential for soil bioremediation. Studies indicate its ability to absorb heavy metals such as iron (Fe), manganese (Mn), zinc (Zn), chromium (Cr), cadmium (Cd), nickel (Ni), and copper (Cu) through its roots, thereby reducing their concentrations in contaminated soils [33,34]. The absorbed metals accumulate in various parts of the tree, including its trunk, as evidenced by analyses of tree rings from specimens grown in areas contaminated by copper smelting [35]. Beyond its ecological impact, *P. juliflora* has significant economic and practical uses. Its wood is highly valued for fuelwood and charcoal production due to its high calorific value. Additionally, the pods of the tree are used as livestock feed, particularly in arid regions where fodder is scarce. The species also shows promise in bioenergy production and is being explored for its potential in ethanol generation [36,37]. However, these benefits are often overshadowed by its invasive nature in non-native regions. Despite its capacity for soil remediation and other ecological benefits, *P. juliflora* poses challenges that cannot be overlooked. Its displacement of native vegetation reduces biodiversity, and its aggressive propagation requires significant management efforts. For example, mechanical removal methods often fail due to the tree’s ability to resprout from stumps and roots, necessitating more expensive and labor-intensive approaches, including chemical treatments [38].

### 3.1. Uses of Prosopis juliflora

*P. juliflora* has been utilized for centuries in various regions worldwide. For example, in India, its pods were consumed, and the seeds were ground into flour for preparing traditional foods like atoles. Wood was employed to craft weapons, while the gum produced by the tree was used in the creation of adhesives and dyes. Similarly, in Mexico, the wood served as a foundation material for houses, and the pods were also a food source. Additionally, the gum extracted from the tree was employed in dye production. In both regions, the tree was highly valued for its medicinal properties, particularly for wound healing [23,36,39]. In modern times, *P. juliflora* has gained attention as a versatile resource. Its biomass is used as a fuel source due to its excellent combustion efficiency, with compounds extracted for biodiesel production [40]. The pods are processed into flours used in the preparation of bread, biscuits, and other foods, and the gums have shown potential to replace guar gum or gum Arabic due to their similar characteristics. The tree also supports apiculture as a source of honey [40]. In livestock farming, pods are a valuable feed for animals such as sheep, cows, and horses, thanks to their high energy, protein, calcium, phosphorus, and sucrose content. However, excessive use must be avoided because the seeds contain alkaloids that can harm the nervous system of animals [24,41]. In addition to these traditional and modern applications, the species plays a crucial role in environmental conservation. Its ability to thrive in arid and semi-arid regions makes it an effective tool for combating desertification and controlling soil erosion. The tree is widely planted as a windbreak to protect crops and as part of agroforestry systems, where it improves soil fertility and provides shade for other plants [36]. Furthermore, *P. juliflora* contributes to reforestation projects aimed at restoring degraded landscapes, especially in regions where native vegetation struggles to survive [42]. In medicine, alkaloids derived from *P. juliflora* have shown potential in the treatment of conditions like Alzheimer’s disease. The plant is also used in traditional infusions to treat injuries and skin conditions and as an expectorant, although improper use or excessive dosages may pose health risks [36]. Additionally, studies indicate that extracts from the pods and leaves have antimicrobial and anti-inflammatory properties, which could be further explored for pharmaceutical applications [39]. Economically, *P. juliflora* provides significant benefits to local communities in arid regions. Its products, including firewood, charcoal, honey, and animal feed, are often sold in local markets, providing a critical source of income. In some areas, the tree’s cultivation has been integrated into community-based management programs aimed at improving rural livelihoods [28].

### 3.2. Phytochemical Compounds of Prosopis juliflora and Their Pharmacological Potential

As shown in Table 1, among the chemical compounds found in *P. juliflora* are flavonoids in the highest concentration, followed by alkaloids, saponins, phenols, and, to a lesser extent, tannins, found in the leaves, which could help us in healthcare due to their antimicrobial, antioxidant, antimalarial, larvicidal, insecticidal, antitumoural, and anthelmintic activities [23]. In addition to their antimicrobial and antioxidant activities, flavonoids have been widely studied for their potential role in preventing cardiovascular diseases and reducing the risk of certain cancers. Their anti-inflammatory properties make them useful in the treatment of chronic inflammatory conditions, further enhancing the pharmacological potential of *P. juliflora* [43]. The compounds are distributed in different ways and in different concentrations; an example of this is the flower extract, where alkaloids, terpenoids, steroids, phenolics, and flavonoids were found, while in the stem, phenolic compounds, flavonoids, terpenes, and steroids were found in low concentrations, while in roots tannins, phenolics, flavonoids, alkaloids, saponin, terpenes, and steroids were obtained. Among the compounds to which pharmacological activities of an anthelmintic type are attributed are alkaloids, tannins, saponins, flavonoids, sterols, and triterpenes [39]. To obtain these extracts from *P. juliflora*, several authors agree that extraction is best carried out using ethanol to obtain a higher concentration of phytochemicals [44,45,46]. Further research has indicated that the alkaloids present in *P. juliflora* may have neuroprotective properties. These compounds could potentially play a role in the prevention or treatment of neurodegenerative diseases, such as Alzheimer’s and Parkinson’s disease, due to their ability to modulate neurotransmitter activity and protect neurons from oxidative stress [47,48]. Another potential of these compounds is their antimicrobial activity due to the alkaloids found in the pods, which are an alternative to reduce the production of methane and carbon dioxide produced by the digestion of ruminants, as they help to reduce the microbial load in their digestive system [49]. It is important to note that the bioactive compounds found in *P. juliflora* may interact with certain pharmaceutical drugs, potentially altering their effects. This highlights the need for caution when using *P. juliflora* extracts in conjunction with other medications [50]. Despite the promising potential of the compounds found in this plant, they must be used with caution. While certain compounds, when consumed in low or controlled quantities, can be beneficial, excessive intake or improper handling can be harmful and even fatal. Wamburu et al. [44] evaluated the toxicity of this plant using Wistar mice which were orally administered certain doses of leaf extract according to their weight; they found that at doses > 250 mg/kg, the mice died, while at that exact dose, only symptoms such as hypoactivity, pilo-erection, loss of appetite, salivation, and hyperventilation manifested themselves at low levels compared to the other groups at higher doses, where deaths occurred.

## 4. Most Common Parasitic Diseases in Equines

**Figure 1:**
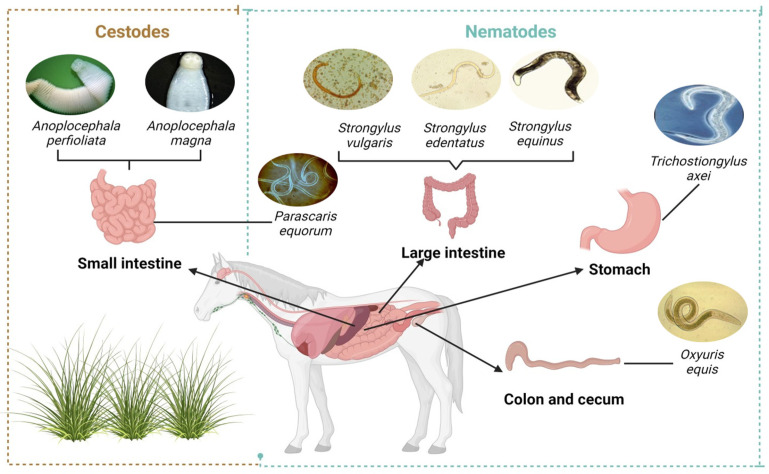
Main gastrointestinal parasites in horses.

The most common parasites found in equines are gastrointestinal parasites such as cestodes *Anoplocephala perfoliata* and *Anoplocephala magna*, and nematodes, mainly *Strongylus vulgaris*, *Strongylus edentatus*, *Strongylus equinus*, *Parascaris equorum*, and *Oxyuris equis*, as shown in Figure 1. The most common symptoms of these parasites are colic, severe weight loss, and diarrhea [11,51].

Depending on the region where the animal is located, it can find more parasitic species, such as those from European regions that show *Strongylus vulgaris*, *Dictyocaulus arnfieldi*, *Oxyuris qui*, *Gasterophilus* spp., *Strongyloides westeri*, *Habronema*, *Draschia* spp., and *Fasciola hepatica* [36]. In tropical regions, equines are often affected by *Trypanosoma spp.*, *Theileria equi*, and *Babesia caballi*, which cause significant health and economic impacts in endemic areas [61]. The detection of gastrointestinal parasites in horses is typically performed using coproparasitological techniques such as the McMaster method, mini-FLOTAC, and fecal cultures. The Faecal Egg Count Reduction Test (FECRT), on the other hand, is specifically used to assess anthelmintic resistance rather than to detect the presence of parasites. However, not all horse owners have access to these diagnostic tools or sufficient knowledge about proper animal and environmental hygiene, which are crucial for preventing and identifying infections in both thoroughbred and working horses [51]. Other diagnostic methods include polymerase chain reaction (PCR) and enzyme-linked immunosorbent assays (ELISAs), which offer higher sensitivity and specificity, especially for subclinical infections [62].

Studies conducted by Peregrine et al. [63] and Königová et al. [64] have reported that prolonged administration of benzimidazole-based antiparasitic drugs at doses of 5–10 mg/kg of body weight can lead to resistance in horses. However, these treatments still contributed to a reduction in helminth egg counts. Further clarification is needed regarding the exact duration of administration and the percentage of reduction achieved. Resistance has also been observed in macrocyclic lactones, with *Parascaris equorum* showing particular resilience, complicating its control in young horses [65]. In another study, they evaluated resistance to tetrahydropyrimidines and benzamidazoles in *Parascaris* sp.; in this study, they discovered new risk factors for parasitosis, such as the movement of horses from stable to stable and the size of the farm where they are raised. They found that the most prone to disease were those with the greatest movement, as well as those from large farms. They also point out the importance of the stress on the animal when it is subjected to more forced work, such as plowing, causing immunosuppression [52]. For the resistance of ivermectin (macrocyclic lactone), it was found to be effective only in mature horses rather than in foals at a dose of 0.2 mg/kg, confirming its effectiveness in them as they have greater exposure to parasites, thus developing greater acquired immunity [54]. Another factor to be evaluated is the route of administration of antiparasitic drugs, for which a study evaluated the oral and intramuscular (IM) routes, finding that the best option is IM, which showed greater effectiveness against parasites than the oral route [66]. Currently, several researchers have proposed that the use of antiparasitic drugs should be very low to avoid greater resistance to them, as well as the rotation of these antiparasitic drugs and the correct selection of treatments depending on the stages of the parasites found (Table 2). Additionally, integrated parasite control strategies are gaining traction, emphasizing the use of pasture rotation, regular deworming schedules tailored to seasonal parasite activity, and the application of biological controls like predatory fungi to reduce larval stages in manure [67].

## 5. Mechanism of Action of Phytochemical Compounds from *Prosopis juliflora* Against Parasites

The mode of action of phytochemical compounds from *Prosopis juliflora* against equine parasites has been extensively explored. Ethanol extracts of *P. juliflora* have demonstrated antimicrobial effects in ruminants and, by extension, could be applied to equines facing similar parasitic infestations. Most studies focus on evaluating the ovicidal effect of *P. juliflora* against various parasites. For instance, one in vitro study reported that a dose of 2 mg/mL of leaf extracts completely inhibited egg hatching in vitro for *Haemonchus contortus*, *Trichostrongylus* spp., and *Oesophagostomum* spp. [76]. In another study, the roots and leaves of the plant underwent phytochemical screening, attributing anthelmintic properties to saponins, tannins, flavonoids, alkaloids, and sterols [39]. Encapsulated leaf extracts exhibited stronger inhibitory activity on helminth egg hatching compared to root extracts, with results comparable to the control drug albendazole. This positions *P. juliflora* as a promising alternative antiparasitic agent for ruminants and equines exhibiting resistance to synthetic drugs [77]. Similarly, Sawal et al. (2018) [24] evaluated the in vitro gastrointestinal anthelmintic activity of pods of *P. juliflora* against *Haemonchus*, *Trichostrongylus*, and *Oesophagostomum* [46]. The study highlighted that alkaloid fractions, particularly juliprosopin, were responsible for significant inhibition of egg hatching, larval migration, and motility. However, while promising, the precise mechanism of action remains under investigation. Alkaloids in *P. juliflora* are believed to inhibit enzymatic activities crucial for degrading the egg membrane [24]. Additionally, saponins destabilize parasite membranes, while tannins inhibit larval release from eggs [78]. Recent research has emphasized the need to assess the broader applicability of *P. juliflora* for equine parasite control in real-world conditions. Studies comparing the efficacy of plant-based compounds to conventional drugs like albendazole have shown encouraging results. The comparable efficacy in inhibiting egg hatching and larval development positions *P. juliflora* as a viable alternative, especially where synthetic drugs fail due to resistance. Moreover, field studies involving infected animals, particularly equines, remain limited [79,80,81,82]. Expanding such research could provide crucial insights into the optimal dosages, long-term safety, and cost-effectiveness of these natural compounds in diverse equine populations.

Economic and ecological considerations further highlight the value of *P. juliflora*. Its widespread availability in arid and semi-arid regions, where resources for synthetic drugs may be scarce, underscores its potential as a sustainable and cost-effective solution. Utilizing a locally abundant resource reduces reliance on expensive imports and supports the ecological balance by managing the often-invasive nature of *P. juliflora* in affected areas [83,84]. Despite its promise, the application of *P. juliflora* in equine parasite management must address certain limitations. While the plant’s toxicity in controlled doses appears minimal, improper handling or excessive consumption of alkaloids can cause adverse effects [85,86]. Therefore, to mitigate risks, it is essential to establish standardized formulations and dosing protocols based on evidence-backed recommendations. Future research should focus on defining optimal dosages, treatment durations, and safety guidelines to ensure efficacy while minimizing resistance development.

In conclusion, the phytochemical compounds of *P. juliflora* present a compelling case for use in equine parasite control. The plant’s antimicrobial, ovicidal, and anthelmintic properties demonstrate considerable promise in addressing parasite resistance to conventional drugs. However, further research is necessary to optimize its practical application, ensuring efficacy, safety, and economic feasibility in diverse equine management systems.

## 6. Possible Adverse Effects of Mesquite

Excessive administration of mesquite (*Prosopis juliflora*) pods and leaves to livestock can result in severe health problems due to the high alkaloid content. These alkaloids, particularly juliprosopine and juliprosin, are neurotoxic and can lead to neurological disorders by affecting neurons and glial cells [41]. Observable symptoms in affected animals include excessive salivation, dehydration, atrophy of the masseter muscles, difficulty in swallowing and chewing, significant weight loss, and, in severe cases, death [41]. The primary alkaloid implicated in such toxicity is juliprosopine, a piperidine alkaloid. Research has shown that extracts of juliprosopine and its fractions act directly on glial cells, causing mitochondrial dysfunction by reducing ATP production [87]. This energy deficit triggers cytotoxicity, neuronal dysfunction, and, ultimately, cell death. The alkaloid’s ability to disrupt mitochondrial activity underscores its potency, even at relatively low concentrations [88]. In conclusion, the adverse effects of mesquite are highly dose-dependent, but the specific toxic dose thresholds remain unclear. These effects are also influenced by the duration of exposure and individual susceptibility. Further research is needed to determine the precise dosage levels at which toxicity occurs in horses. While mesquite has many beneficial properties, its use in animal feed should be carefully monitored to avoid toxicity. Future studies should aim to identify safe thresholds for mesquite consumption and explore potential mitigation strategies, such as detoxification processes, to reduce the alkaloid content before its use as a feed additive.

## 7. Conclusions

Current research suggests that natural compounds, such as those derived from *Prosopis juliflora*, may offer an alternative to synthetic antiparasitic drugs, particularly due to their complex mechanisms of action that could help mitigate resistance. However, while *P. juliflora* has demonstrated antiparasitic properties in other species, but its efficacy, specifically against equine gastrointestinal parasites, remains largely unexplored. Further targeted research is essential to determine its potential role in equine parasite management, including in vivo studies to establish safe and effective therapeutic dosages. Mesquite’s phytochemicals (alkaloids, saponins, and tannins) exhibit ovicidal and larvicidal properties, suggesting potential applications in parasite control. Optimizing extraction, formulation, and encapsulation techniques could enhance bioavailability while minimizing toxicity. However, these effects must be validated in equines before recommending its inclusion in veterinary protocols. Comprehensive parasite management should integrate both conventional and potential natural alternatives, emphasizing routine fecal egg count monitoring, strategic deworming, and improved pasture management. Additionally, educating horse owners and caretakers on best practices is crucial for sustainable parasite control. In conclusion, *Prosopis juliflora* holds potential as an antiparasitic agent, but its specific efficacy against equine gastrointestinal parasites requires further investigation. A balanced approach that combines ongoing research, responsible use of natural compounds, and evidence-based veterinary practices will be key to sustainable equine parasite management. Although plant-derived compounds are increasingly recognized as promising alternatives to conventional anthelmintics, the potential for the development of resistance associated with their use warrants careful consideration. Currently, there is a paucity of the scientific literature specifically addressing this issue. Nonetheless, given the bioactive nature of many phytochemicals and their potential to exert selective pressure on parasitic populations, the emergence of resistance cannot be completely excluded. Further investigations are required to elucidate the possible long-term effects of repeated exposure to plant-based anthelmintics and to determine whether such compounds may contribute to resistance mechanisms.

## Figures and Tables

**Table 1:** Main phytochemical compounds of *Prosopis juliflora* and their functions.

Phytochemical Compound	Concentration (% on DM)	Location	Examples	Function	Reference
Alkaloids	~3.6	Leaves, Pods, Seeds	Juliprosin, Juliflorin, Isojuliprosin, Piperidine, Juliflorin (juliprosopina), julifloricin, julifloridine	Antimicrobial activity, Cytotoxicity, improved digestion in livestock, neuroprotective potential	[51]
Flavonoids	~16	Leaves, Flowers, Bark	4′-O-methylgallocatechin, Mesquitol	Antioxidant, Anti-inflammatory, Cardioprotective, Cancer prevention, Neuroprotective	[52]
Saponins	~2.2	Leaves, Root	Aescin, Dioscin	Antimicrobial, Antioxidant, Antimalarial, Insecticidal	[53]
Phenols	~0.66	Leaves, Pods, Flowers	Affeic acid, chlorogenic acid	Antioxidant, Anti-inflammatory, Anticancer	[53]
Tannins	~0.33	Roots, Leaves	Catechin, Epicatechin	Antimicrobial, Antioxidant, Anti-inflammatory, Anticancer	[54]
Terpenoids	~1.0	Leaves, Flowers	Limonene, Beta-caryophyllene	Anti-inflammatory, Anticancer, Antimicrobial	[43,55]
Sterols	~0.5	Leaves, Stem	β-sitosterol, Stigmasterol	Antiinflammatory, Cardiovascular protection	[56,57]
Lignans	~0.2	Pods, Seeds	Secoisolariciresinol, Matairesinol	Antioxidant, Antitumoral, Hormonal balance	[58,59]
Acidic phenols	~0.2	Leaves, Pods	Caffeic acid, Ferulic acid	Antioxidant, Anti-inflammatory	[60]
Glycosides	~0.3	Leaves, Pods	Stevioside, Aucubin	Antidiabetic, Antiallergic, Antioxidant	[59]

**Table 2:** Antiparasitics commonly used to treat different gastrointestinal infections in horses.

Chemical Compound	Examples	Concentration	Impact as an Antiparasitic	Resistance	Reference
Macrocyclic lactones	Ivermectin	0.2 mg/kg	Decreased egg count	Present resistance, controlled with rotation	[54,66,68]
	Moxidectin	0.4 mg/kg	Effective against larvae stages	-	[54]
	Doramectin	0.2 mg/kg	Targeted strongyles and ascaridis	Emerging resistance	[51]
Tetrahydropyrimidines	Pyrantelpamoate	19 mg/kg	Effective against adult strongyles	Present resistance in certain populations	[52,65,69]
Benzidamizoles	Fenbendazole	5–10 mg/kg	Reduced strongyle egg counts	Present resistance, controlled by rotation	[52,69,70,71]
	Oxibendazole	7.5 mg/kg	Reduced Parascaris equorum eggs	Resistance in foals	[52,72]
	Albendazole	7.5 mg/kg	Broad-spectrum efficacy	Emerging resistance in some regions	[73]
Organophosphates	Trichlorfon	25 mg/kg	Effective against bots	Limited resistance reported	[74]
Phytochemical-based antiparasitics	Neem oil	1–5% solution	Inhibits larval development	No resistance observed yet	[75]

## Data Availability

Not applicable.
